# Financing costs and the competitiveness of renewable power

**DOI:** 10.1016/j.isci.2025.113777

**Published:** 2025-10-15

**Authors:** Christian Wilson, Gireesh Shrimali, Ben Caldecott

**Affiliations:** 1 Smith School of Enterprise and the Environment, School of Geography and the Environment, University of Oxford, Oxfordshire OX1 3QY, UK

**Keywords:** Applied sciences, Energy engineering, Energy Modeling

## Abstract

Relative to fossil fuels, the cost of renewables is more sensitive to the cost of capital (CoC). Here, we analyze the impact of changing financing costs on the competitiveness of renewable power. Following a decade of low rates, interest rate rises occurred in developed and developing economies from 2022. In the U.S., this added 9% to the levelized cost of electricity (LCOE) of combined cycle gas turbines, compared to 18% for solar photovoltaics (12% with tax credits). At present, reductions in CoC would have limited impact on competitiveness in Europe, given high fuel and carbon prices, but in the U.S., China, and India, reductions can facilitate convergence in LCOE between certain renewable technologies and lower-cost fossil fuel power. Consequently, policies reducing renewable CoC can accelerate cost parity with fossil fuels. However, policies that increase fossil fuel CoC are less effective, given the lower sensitivity to financing costs.

## Highlights

• Renewable power is more sensitive to the cost of capital (CoC) than fossil fuels
• Policies reducing the upfront costs of renewables lower the sensitivity to CoC
• In certain regions, renewables remain more expensive than fossil fuels
• Lower financing costs can result in cost parity between renewables and fossil fuels

## Graphical abstract

**Figure:**
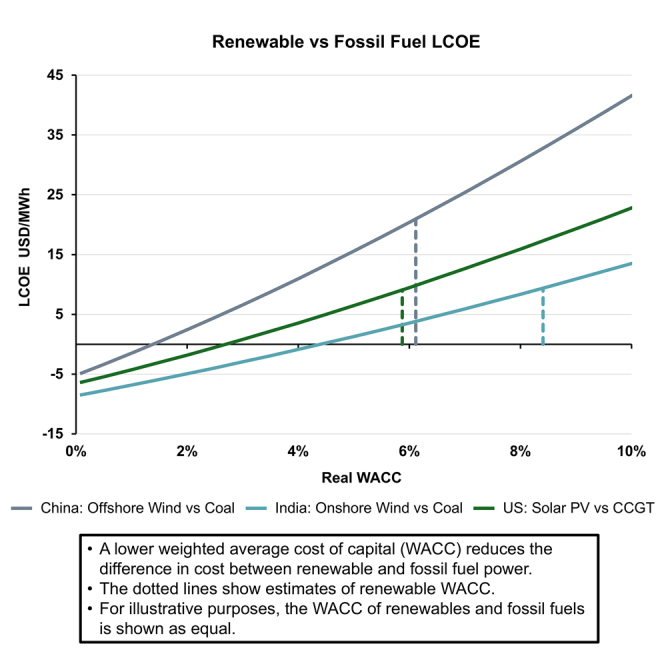

## Introduction

Decarbonizing power generation is critical, as the sector accounts for 40% of global greenhouse gas emissions.^1^ To limit global warming to 2°C, renewables need to exceed 80% of power generation by 2050, of which 82% is in solar and wind.^1^^,^^2^ This requires low-carbon investment to double,^3^^,^^4^ making the weighted average cost of capital (WACC) of renewables a key driver of power decarbonization.^5^^,^^6^ As renewable power assets have a higher capital intensity than fossil fuels, with significant upfront capital costs and limited operational costs, the levelized cost of energy (LCOE) of renewables is more sensitive to changes in WACC.^7^^,^^8^^,^^9^ Consequently, alongside supportive policies, technological advancements, and high learning rates,^10^ falls in interest rates over the past two decades have acted as a tailwind for renewables, lowering the WACC, and therefore, improving competitiveness relative to fossil fuels.^11^ Despite this, in regions such as the US, India, and China, fossil fuel power remains competitive or indeed cheaper than either solar or wind power.

In recent years, interest rates have increased sharply to combat inflation stoked by an economic recovery post-COVID-19 and an energy crisis prompted by Russia’s invasion of Ukraine, with the median advanced economy central bank rate rising from close to zero in 2021 to 5% in 2024.^12^ A 2019 study outlined such an increase as an “extreme scenario” with negative implications for the levelized cost of energy (LCOE) of renewables.^13^ This extreme scenario has come to pass, raising the question of what impact it has had and what will happen if rates now fall. Indeed, in 2024, U.S. and European Union (EU) central banks cut interest rates, with continued reductions expected.^12^ Whether future changes in financing costs materially impact the cost competitiveness of renewables relative to fossil fuels will depend on regional dynamics. An assessment of these dynamics can help policymakers and multilateral development banks identify where reductions in WACC are most needed.

To address these issues, we carry out the following analyses. First, we utilize asset-level project finance transactions to track and compare the cost of debt of renewables and fossil fuel power between 2000 and 2025. Second, we examine the impact of recent increases in interest rates on the LCOE of renewables without battery storage and fossil fuel power assets in North America. Third, we model the effect of changes in financing costs on the cost competitiveness of renewables without battery storage relative to fossil fuels across regions, including Europe, the U.S., China, and India. The methods section is structured along these steps.

Following the aftermath of the 2008 financial crisis, we find that lending spreads for renewable and fossil fuel power assets have compressed following unconventional monetary policy,^14^ but between renewables and fossil fuels, there has been a gap in spreads of around −100 basis points (bps), indicating that renewables are perceived as lower risk. A basis point equals a 100^th^ of a percent. However, while spreads have fallen, recent interest rate rises have sharply increased overall financing costs. In North America, the nominal cost of debt for solar and wind transactions increased from less than 2% in 2021 to 6.5% in 2024.

These changes have impacted renewables more than fossil fuels. In the U.S., higher financing costs added 18% to the LCOE of solar PV without tax credits, while adding only 9% to the LCOE of combined cycle gas turbines (CCGT). With tax credits in the Inflation Redution Act (IRA), financing costs added 12% to solar PV LCOE, due to reductions in capital costs, which, in turn, reduces sensitivity to WACC. Going forward, the impact of changes in WACC differs by region. In Europe, reductions in WACC do not make a material difference in cost competitiveness, as renewables are already significantly cheaper than fossil fuels. However, in the U.S., a 25% fall in solar PV WACC reduces the gap in LCOE with CCGT from +9.1 USD/MWh to 2.4 USD/MWh. A 25% reduction in India effectively results in cost parity between onshore wind and coal, while in China, the difference in LCOE between offshore wind and coal is reduced by 37%. These results show how changing financing costs can trigger points in the cost competitiveness of renewables. Following this analysis, we outline implications for policy and future research.

## Results

### Risk of renewables versus fossil fuels

To track asset-level financing costs, we extract 8,144 project finance debt transactions in the power sector between 2000 and September 2025 from LSEG Data and Analytics. Transactions are priced as a credit spread over floating interest rates. As nearly all project finance transactions are non-recourse, with the asset as collateral, the spread compensates the lender for the asset-level credit risk they are exposed to. Spread data are available for 1,649 transactions, of which 29% are wind, 22% are solar, 28% are gas, 5% are coal, and 15% are other energy types. In this analysis, other energy types are excluded, limiting the table to 1,385 transactions (see Figure S1 for a full breakdown).

**Figure 1:**
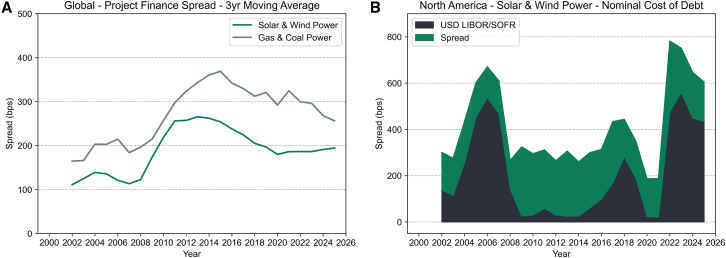
Renewable and fossil fuel spread over time (A) Three-year moving average of project finance loan spreads for global solar and wind and gas and coal power assets. (B) Nominal cost of debt for solar and wind project finance transactions in North America, broken down in average annual spread and the reference floating interest rate (3-month US Dollar London Interbank Offered Rate (LIBOR) and Secured Overnight Financing Rate (SOFR)). 2025 data ends in August. See Figures S1–S4, for information regarding the distribution of the data and specific technologies.

Figure 1 shows the average solar and wind and gas and coal spreads over time (see STAR Methods). Spreads increased during the 2008 financial crisis, followed by a decline after 2013. Solar and wind had lower spreads than fossil fuels before 2008, after which this gap increased to around 100 bps. In recent years, there have been indications of rising spreads for solar and wind, reflecting challenging conditions with increasing input costs and supply chain issues.^15^ We group solar and wind given similarities in spread (see Figure S2).

Figure 1 shows that renewables have historically had lower financing costs than fossil fuels, reflecting the fact that, unlike fossil fuels, solar and wind have no fuel price risk, and that power is usually sold at fixed prices via feed-in tariffs or auctions. However, other factors could drive divergence, such as loan size, maturity, credit rating of the project sponsor, and country-level conditions. In Table 1, we tabulate results from an OLS regression controlling for asset and country-level characteristics, along with year, country, and currency fixed effects (see Tables S1 and S2 for variable definitions and summary statistics).

**Table 1:** Regression of renewable versus fossil fuel spreads

	All Transactions	2000–2010			2011–2024		
		All	US	ex-US	All	US	ex-US
	1	2	3	4	5	6	7
Renewable Asset	−83.133^∗∗∗^	−53.217^∗∗∗^	−108.380^∗∗∗^	4.453	−106.242^∗∗∗^	−115.675^∗∗∗^	−83.380^∗∗∗^
	(8.061)	(12.944)	(13.148)	(21.139)	(10.344)	(11.025)	(24.700)
Year FE	Yes	Yes	Yes	Yes	Yes	Yes	Yes
Country FE	Yes	Yes	Yes	Yes	Yes	Yes	Yes
Currency FE	Yes	Yes	Yes	Yes	Yes	Yes	Yes
Asset-Level Controls	Yes	Yes	Yes	Yes	Yes	Yes	Yes
Country-Level Controls	Yes	Yes	Yes	Yes	Yes	Yes	Yes
N	1198	529	177	352	669	315	354

For the coefficients of the ordinary least squares regression, ^∗^*p* < 0.10, ^∗∗^*p* < 0.05, ^∗∗∗^*p* < 0.01 denote statistical significance at the 10%, 5%, and 1% level, respectively. Robust standard errors are shown in parentheses. See Tables S1 and S2 for variable definitions and summary statistics. Table S3 shows results for solar and wind separately.

Using a dummy variable to indicate when an asset is renewable, we observe that renewables have lower spreads by 83 bps over the full period. These results hold if limited to solar or wind, at −82 bps and −88 bps, respectively (see Table S3). For wind, this relationship holds in both decades of the sample, but only in the second decade for solar, corresponding to the divergence shown in Figure 1. In the final sample, 82% of transactions are in OECD countries and 39% are in the U.S. We split the sample accordingly. In the U.S., there is evidence that renewables have lower spreads in both time periods, but outside of the U.S., this only holds during 2011–2024.

A lower spread for renewables indicates that policies such as feed-in tariffs and Renewable Portfolio Standards (RPS) have successfully reduced risk, while carbon prices risk could have had the opposite effect for fossil fuel power. Lower spreads reduce the WACC; however, recent interest rate rises have offset this decrease, leading to a sharp increase in the overall cost of debt for new renewable assets (see Figures 1B and S4 for gas & coal).

### Changing interest rates

To examine the impact of rising interest rates on power generation costs, we use the U.S. as a case study. While other large markets, such as the EU, have also experienced sharp rises in interest rates, renewables are significantly cheaper than fossil fuels, making financing costs less relevant to competitiveness (discussed in the following section). To model U.S. LCOE, we utilize the technology-specific cost and performance parameters for utility-scale assets from the 2024 and 2022 Electricity Annual Technology Baseline (ATB) published by the National Renewable Energy Laboratory (NREL)^16^^,^^17^ (see Table S4). We focus on utility-scale renewables to aid comparison with fossil fuels. To note, smaller assets may have higher financing and construction costs per MW. In line with NREL, we model an increase in real interest rates from −0.5% to 2.5% between their 2020 and 2024 reports, with a gap in spreads between renewables and fossil fuels of −100 bps, corresponding to Table 1. In line with NREL, the International Renewable Energy Agency (IRENA),^18^ and the International Energy Agency (IEA),^19^ we use a real rather than nominal WACC to adjust for the effects of inflation on costs.

**Figure 2:**
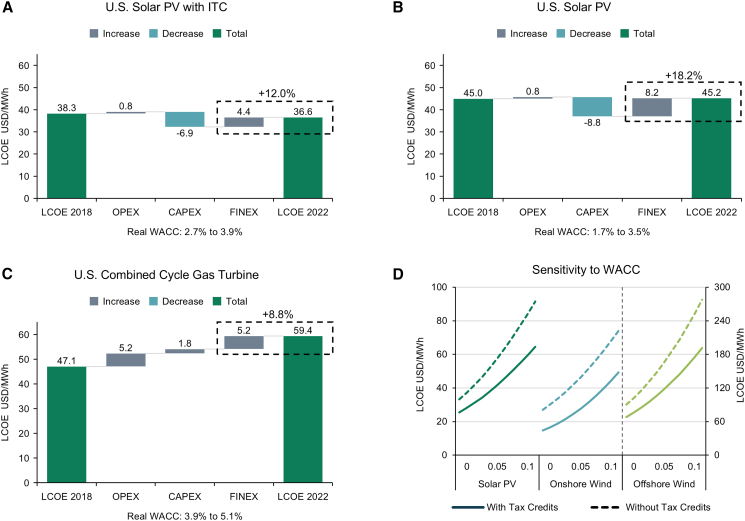
Historical change in LCOE (A–C) Change in LCOE between the NREL 2020 ATB model (base year 2018) and the NREL 2024 ATB model (base year 2022) is broken down into the change in OPEX, CAPEX, and FINEX for solar PV with investment tax credits (ITC) (A), solar PV without investment tax credits (B), and combined cycle gas turbine (C). (D) The sensitivity of LCOE to WACC with and without tax credits. Solar PV and onshore wind are shown with the left-hand *y* axis. Offshore wind with the right-hand *y* axis. Investment tax credits are applied to solar PV and offshore wind. Production tax credits are applied to onshore wind. See Figure S5 for additional technology types. See Table S4 for technology cost assumptions.

We split changes in LCOE into operating expenditure (OPEX), capital expenditure (CAPEX), and financing costs (FINEX) (see STAR Methods for details). We use solar PV without battery storage as an example, as this renewable technology is closest to CCGT in costs. Figure 2A shows that with tax credits, higher financing costs added 4.4 USD/MWh to LCOE, equivalent to 12.0%. Without these tax credits, 8.2 USD/MWh was added, equivalent to 18.2%. Charts for onshore and fixed bottom offshore wind are shown in Figure S5. For combined cycle gas turbine (CCGT) plants, financing costs added 5.2 USD/MWh to LCOE, equivalent to 8.8%. Without tax credits, the impact of financing costs on LCOE for solar PV was twice that of CCGT. However, investment tax credits (ITC) reduce upfront capital costs, lowering sensitivity to real WACC, as shown in Figure 2D for solar PV and offshore wind. Production tax credits (PTC), which reduce costs for the first 10 years of a project, have a similar but weaker effect on onshore wind.

**Figure 3:**
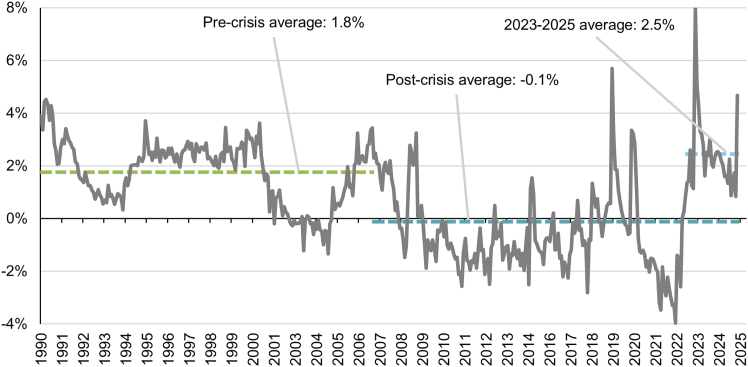
1-Year U.S. real interest rates Pre-crisis refers to before 2007.

**Figure 4:**
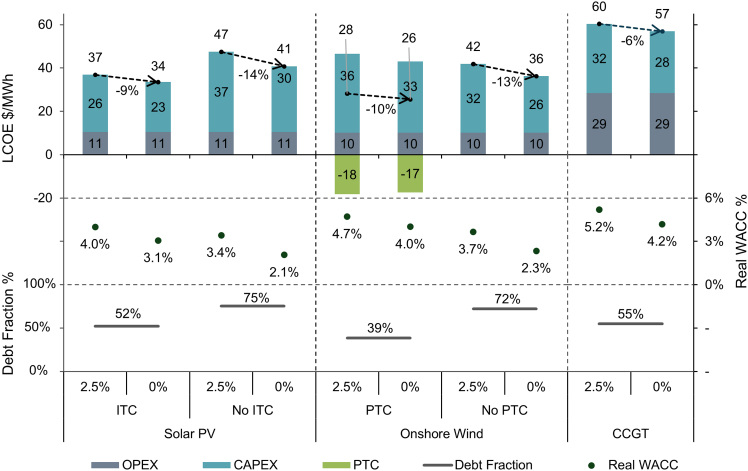
Impact of lower real interest rates The upper panel shows the change in LCOE due to a fall in real interest rates from 2.5% to 0%, with and without tax credits. All other cost and financial assumptions are taken from the NREL 2024 ATB model. LCOE is broken down into OPEX, CAPEX, and the impact of ITC/PTC. See STAR Methods for associated calculations. The middle panel shows the change in real WACC due to changes in real interest rates. The bottom panel shows the debt fraction of financing. See Table S4 for technology cost assumptions.

In the U.S, real interest rates averaged −0.1% after the financial crisis but averaged 2.5% in 2023-2025, in line with the 2024 NREL model estimate of 2.5%^20^ (Figure 3). In Figure 4, we model the impact of a fall in real interest rates from 2.5% back to zero, focusing on solar PV and onshore wind as the cheapest renewables and CCGT as the cheapest fossil fuel. For CCGT, this would reduce LCOE by 6%, reflecting the large share of OPEX in LCOE. Without tax credits, LCOE would fall by 14% and 13% for solar PV and onshore wind. With tax credits, LCOE would fall by 9% and 10%, respectively, reflecting the reduction in upfront costs (during construction or over 10 years) and the lower debt fraction as a result of tax equity financing, which limits the change in real WACC from interest rates.

### Regional financing costs and cost competitiveness

While renewables are more sensitive to financing costs, whether reductions alter cost competitiveness relative to fossil fuels will differ by region. To highlight this, we model LCOE as a function of financing costs in Europe, the U.S., China, and India, which together account for 70% of global power capacity.^1^ We use 2023 technology cost assumptions from the IEA^19^ and 2023 real WACC estimates from IRENA^18^ (see Tables S5–S7, for inputs). While IEA cost assumptions are less detailed than NREL, they are standardized across regions, facilitating comparison.

**Figure 5:**
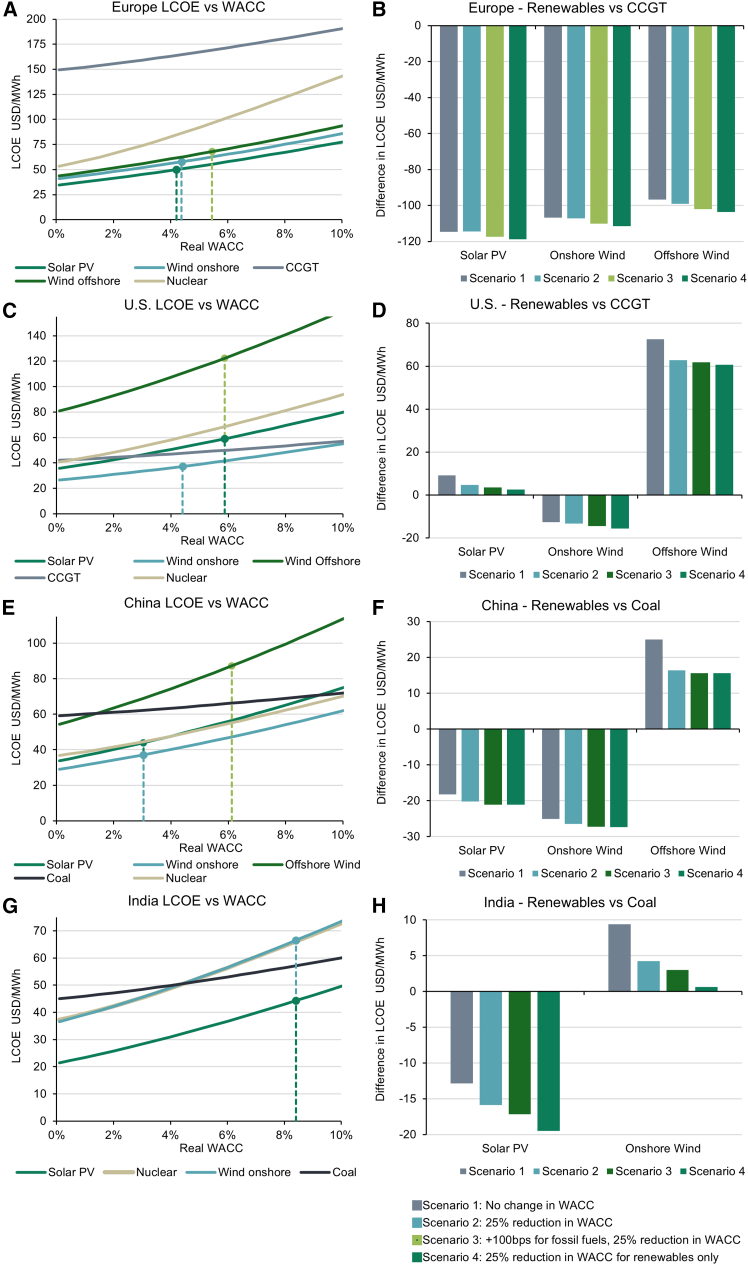
Cost of renewables versus fossil fuels by region (A–H) Left-hand charts show LCOE as real WACC changes. The dotted lines refer to IRENA’s 2023 WACC estimates. Right-hand charts show the difference in LCOE between renewables and fossil fuels as WACC changes. This is shown for Europe (A and B), the U.S. (C and D), China (E and F), and India (G and H). See Tables S5–S7, for technology cost and cost of capital assumptions.

In Figure 5, for each region, we plot LCOE as WACC changes for renewable, nuclear, and fossil fuel power (see STAR Methods). These charts provide insights into the sensitivity of LCOE to WACC across technologies. For a given level of WACC, one can compare the LCOE of different technologies. However, in reality, WACC is not uniform across technologies, due to different risk premiums and capital structures as highlighted in Figures 2 and 4.

In Figure 4, we also calculate the difference in LCOE between renewables and fossil fuels in four scenarios. As IRENA does not provide WACC estimates for fossil fuel power, we have to make assumptions. In scenario 1, we input IRENA’s 2023 WACC figures and assume that fossil fuel WACC equals that of the riskiest renewable technology. In scenario 2, we input a 25% reduction in WACC from scenario 1. In scenario 3, we input a 25% reduction in WACC, after which fossil fuel WACC is 100 bps higher than the riskiest renewable technology. In scenario 4, we input a 25% reduction in renewable WACC and hold fossil fuel WACC constant at the levels used in scenario 1.

This analysis shows that in Europe, renewables are cheaper than CCGT at all levels of WACC modeled (Figure 5A). As a result, while falls in WACC reduce costs for renewables, they have a limited impact on the cost differential with CCGT (Figure 5B).

In the U.S., without tax credits, while onshore wind is cheaper than CCGT at all levels of WACC, at current WACC estimates, solar PV has an LCOE +9.1 USD/MWh higher than CCGT (Figures 5C and 5D). This gap falls to +4.7 USD/MWh and +2.4 USD/MWh in scenarios 2 and 4, respectively. In contrast, offshore wind remains more expensive across scenarios, reflecting the high levels of CAPEX required. In scenario 4, the cost difference with CCGT falls by 16% to +60.6 USD/MWh.

The cheapness of CCGT in the U.S. relative to Europe reflects differences in fuel costs and carbon prices. To calculate OPEX, the IEA technology cost assumptions for fuel, CO2 and operation and maintenance costs are averaged over 10 years. For Europe, carbon prices are assumed to reach 120 by 2030 versus zero for the U.S., while European gas prices are expected to fall from 32.3 USD/MBtu in 2022 to 6.9 USD/MBtu in 2030, compared to 5.1 USD/MBtu to 4.0 USD/MBtu for the U.S. While the 2022 European energy crisis led to a sharp spike in gas prices, the effect of this is mitigated as gas prices are averaged over 10 years. Furthermore, if we assume that gas prices in Europe were equal to those of Japan and China in 2022 using IEA data (14.7 USD/MBtu), the LCOE of CCGT falls by approximately 10%, still well above renewables.

In China and India, coal is the cheapest fossil fuel power. In China, solar PV and onshore wind have a low WACC of 3.04%, reflecting the scale of the domestic industry,^1^ low central bank rates,^21^ and supportive lending practices.^22^ With LCOE already significantly lower than coal, a 25% reduction in WACC has a limited impact on competitiveness (Figure 5F). In contrast, offshore wind has a WACC double that of onshore wind at 6.12%. In scenario 4, a 25% decrease in WACC reduces the difference in LCOE with coal from +24.9 USD/MWh to +15.6 USD/MWh, a significant 37% reduction.

In India, renewable WACC is significantly higher than in other regions at 8.41% (Figure 5G). Yet, solar PV is cheaper than coal at all levels of WACC, reflecting supportive subsidies and renewable energy obligations.^23^ Here, a 25% reduction in WACC in scenario 4 extends the cost differential from −12.8 USD/MWh to −19.5 USD/MWh (Figure 5H). While onshore wind has a higher LCOE than coal in scenario 1, this gap is halved in scenario 2 and effectively eradicated in scenario 4. Given the lack of domestic deployment, offshore wind is not shown in India.

In summary, for select regions and technologies (U.S. solar PV, Chinese offshore wind, and Indian onshore wind), targeted policy interventions that reduce WACC can improve competitiveness relative to fossil fuels. Furthermore, reductions in WACC that impact both renewables and fossil fuels, for example, as a result of interest rate changes, can also close the gap in competitiveness.

For comparison, with regard to nuclear power, due to high capital intensity, the gradient of LCOE curves is comparable to solar PV in China, onshore wind in India, and solar PV in the U.S. In Europe, where nuclear power is more expensive than renewables, LCOE is even more sensitive to WACC.

## Discussion

This paper makes several contributions. While low-carbon firms have been shown to have a lower CoC,^24^^,^^25^^,^^26^^,^^27^^,^^28^ within the power sector, comparisons are challenging as many firms operate low- and high-carbon assets.^29^ Indeed, studies comparing low- and high-carbon energy firms have excluded power.^30^ By focusing on assets rather than firms, we show that renewables have lower spreads than fossil fuels. While our sample was concentrated in developed economies, with further research needed in developing economies, the findings are relevant to researchers modeling the impact of financing costs on the energy transition. As renewables tend to use project finance and fossil fuels tend to use corporate finance,^31^ studies have estimated renewable WACC at the asset level and fossil fuel WACC at the firm level, resulting in a higher WACC for renewables versus gas.^32^^,^^33^ As diversified firms are lower risk than a single asset, this approach could underestimate fossil fuel WACC, especially if firms adjust discount rates when assessing project-specific risk.^34^ As a result, future work should evaluate the impact of divergence and convergence in spreads between renewables and fossil fuels.

This paper then documents the outsized impact of recent interest rate rises on the LCOE of renewables without battery storage versus fossil fuels in the U.S., with higher financing costs offsetting lower technology costs. While tax credits in the IRA have accelerated low-carbon investment in the U.S.,^4^ higher interest rates could reduce its effectiveness. Indeed, higher financing costs are cited as a factor for a surge in project cancellations, particularly for offshore wind.^35^^,^^36^ However, we show that tax credits not only reduce LCOE, but also reduce sensitivity to financing costs. As a result, the impact of rising interest rates on competitiveness relative to fossil fuels is more muted than in past studies.^13^ Fiscal support should, therefore, be considered alongside monetary instruments as a mechanism to shield renewables from interest rate rises.^37^ An example of monetary policies for this purpose is the People’s Bank of China’s Carbon Emissions Reduction Facility, which allows banks to borrow at rates of 1.75% for 60% of the value of green loans^38^ compared to over 3% for normal loan rates,^21^ with this facility used to fund renewables.^39^

Lower financing costs for renewables are crucial for improving the economic^40^ and political viability of the energy transition.^41^ This paper shows how lower financing costs reduce the cost of renewables (without battery storage) and facilitate cost parity with fossil fuels—a potential tipping point in energy systems.^42^^,^^43^^,^^44^ However, there is significant regional variation. In Europe and China, falls in WACC have a limited impact on competitiveness, whereas in the U.S. and India, falls in WACC have the potential to result in cost parity with fossil fuels for solar PV and onshore wind, respectively. In particular, in India, high financing costs remain a problem, increasing the LCOE of renewables and the reliance on subsidies.^45^ Here, interventions by policymakers and development banks can target investor concerns regarding regulatory, currency, and off-taker risk.^45^ For researchers, these findings reinforce the need to account for regional variations in financing costs when modeling the energy transition. Historically, models have assumed a uniform WACC,^46^^,^^47^ but when regional variations are accounted for, this leads to major differences in the future deployment of renewables.^32^^,^^33^^,^^48^

While falls in the cost of renewables are critical, this alone is not enough to decarbonize the power sector. In China, the U.S., and India, fossil fuel power remains cheap, with new capacity additions continuing at pace^49^^,^^50^^,^^51^ and risking the overshoot of climate goals.^52^ In contrast, in the EU, where fossil fuel power costs are higher due to fuel and carbon prices, new capacity additions have been more limited.^51^^,^^53^ Even if stranded asset risks are priced in the CoC,^52^^,^^54^^,^^55^^,^^56^ low sensitivity to financing costs will mute any impact on fossil fuel LCOE. As a result, policies that increase the cost of fossil fuels, such as carbon prices, have an essential role in conjunction with policies that reduce the risk and costs of renewables.^57^ Yet, even with higher costs, demand for fossil fuel power is expected as governments balance intermittent renewables^58^ and data centers seek out predictable generation.^59^ In this context, battery storage and nuclear can displace fossil fuels as a baseload power source.^60^^,^^61^ Given the capital-intensive nature of these technologies, reducing financing costs will be critical.

### Limitations of the study

While this study provides important insights, there are limitations worth considering. When analyzing project finance data, we are limited by the scope of the sample, as only certain countries disclose spreads at the transaction level, skewing the sample toward developed countries, in particular, the U.S. and Europe. Furthermore, by technology type, a low proportion of transactions provide spread data, as shown in Figure S1. To combat this, we include additional asset-level and country controls, as well as country fixed effects. However, we cannot rule out that our results are biased by the types of countries and transactions that disclose spreads. For example, borrowers may be incentivized to disclose spreads if they are more favorable. When analyzing the impact of changing financing costs on the competitiveness of renewables in different regions (Figure 5), data from the IEA on LCOE cost components is utilized, as it is provided in a standardized manner. However, in comparison to the NREL data used to analyze the U.S. (Figure 2), less detail is provided. For example, NREL breaks down OPEX and CAPEX into sub-components and provides clarity on all underlying assumptions, such as the capital structure. Similarly, we utilize IRENA data that provides country-level estimates of the CoC of specific renewable technologies. However, this data are not provided for fossil fuels, meaning that assumptions are made, as detailed in scenarios 1–4 in Figure 5. If more detailed datasets covering multiple regions become available, this would improve the accuracy of the analysis presented. Finally, while we focus on the impact of financing costs, other factors, such as fuel prices, carbon prices, and technology costs, drive changes in LCOE. An in-depth analysis of these factors and how they interact with financing costs could be addressed in future work.

## Resource availability

### Lead contact

Further information and requests for resources and materials should be directed to and will be fulfilled by the lead contact, Christian Wilson (christian.wilson@smithschool.ox.ac.uk).

### Materials availability

This study did not generate new unique reagents.

### Data and code availability

• All data sources used in this study have been reported in Table S8. All data are publicly available except for project finance data, where a license agreement is required with LSEG Data & Analytics.
• Stata code to produce Tables 1 and S3 is available at https://doi.org/10.5281/zenodo.14509412.
• Any additional information required to reanalyze the data used in this paper is available from the lead contact upon request.

## Acknowledgments

This work was supported by the Sunrise Project.

## Author contributions

Conceptualization, C.W., G.S., and B.C.; investigation, C.W. and G.S.; project administration, C.W. and G.S.; visualization, C.W.; resources, C.W. and G.S.; writing—original draft, C.W.; writing—review and editing, C.W., G.S., and B.C.; supervision, G.S. and B.C.

## Declaration of interests

The authors declare no competing interests.

## STAR★Methods

### Key resources table

**Table:** 

REAGENT or RESOURCE	SOURCE	IDENTIFIER
**Deposited data**		

All the data sources used in the study have been reported in the supplemental information (Table S8)	–	N/A

**Software and algorithms**		

Python programming language, version 3.8.10	Python Software Foundation	https://www.python.org/
STATA SE, version 18	StataCorp	https://www.stata.com/

### Method details

#### Project finance transactions

Our analysis begins with analysing project finance transactions in the power sector. These transactions are downloaded from LSEG Data & Analytics using the Screener function from 2000 to August 2025. Transactions are filtered to include those labelled as financed solar, wind, gas, combined cycle turbines, and coal. Solar and wind are grouped due to similar spreads, while gas and coal are grouped as there are insufficient transactions to plot coal separately (see Figures S1 and S2). Over time, annual project finance loan volume for solar and wind has increased from $0.1bn in 2000 to $65.2bn in 2024, while volumes for gas and coal power have declined from $16.1bn to $7.9bn (see Figure S6). Financing costs for these transactions are quoted as a spread over a floating interest rate. For each project finance transaction with multiple tranches, the associated loan amount is calculated as the sum of the value of each tranche. As loan amounts are missing for certain tranches, transaction spread is calculated as a simple average of the underlying tranches rather than a weighted average.

In Figure 1B, we estimate the nominal annual cost of debt for new project finance transactions for solar and wind, broken down into the average annual spread and USD LIBOR/SOFR (Equation 1). The publication of USD LIBOR ended in September 2024, with USD project finance transactions shifting to Secured Overnight Financing Rate (SOFR) as the default replacement rate in 2022.^62^ As shown in Figure S3, USD LIBOR and SOFR closely track each other. Therefore, we replace LIBOR with SOFR after replacement.

(Equation 1) NominalCostofDebt=Spread+FloatingInterestRate

For details on the empirical analysis of project finance spreads, see the STAR Methods section quantification and statistical analysis.

#### Decomposing changes in LCOE

To identify how changes in financing costs have affected the LCOE of renewables and fossil fuel technologies, we utilise the technology-specific cost and performance parameters from the 2024 and 2020 Electricity Annual Technology Baseline (ATB) published by NREL.^16^^,^^17^ These annual reports provide detailed cost assumptions for different technologies in the United States, with a base year of 2022 and 2018. As a result, the NREL 2024 and 2020 models allow us to capture LCOE before and after interest rate rises.

Here, we summarise the NREL LCOE methodology used.^63^ In Equation 2, LCOE is calculated as a function of the capital recovery factor (CRF), project finance factor (ProFinFactor), construction financing factor (ConFinFactor), overnight capital costs (OCC), grid connection costs (GCC), fixed operating costs (FOM), capacity factor (CF), variable operating costs (VOM), fuel costs (FUEL) and production tax credits (PTC).

(Equation 2) $$Levelised\;Cost\;of\;Electricity\;\left(LCOE\right)=\frac{\left(\left(CRF\times ProFinFactor\times ConFinFactor\times \left(OCC+GCC\right)\right)+FOM\right)*1000}{CF\times 8760}+VOM+FUEL-PTC$$

CRF captures the amount needed to break even on an investment and is calculated as the ratio of a constant annuity to the present value of that annuity for a set amount of time *t* (Equation 3). CRF is calculated using real WACC, defined in Equation 4, with nominal WACC defined in Equation 5. Nominal WACC is a function of the debt fraction (DF), real return on equity (RROE), real interest rate (IR) and the tax rate (TR). Nominal to real values are calculated by dividing one plus the relevant discount rate *d* by one plus inflation *i* (Equation 6).

(Equation 3) $$Capital\;Recovery\;Factor\;\left(CRF\right)=WACC\times \frac{1}{(1-\frac{1}{{\left(1+WACC\right)}^{t}}}$$

(Equation 4) $$Real\;Weighted\;Average\;Cost\;of\;Capital\;\left(WACC\right)=\frac{1\;+\;WACC\;Nominal}{1+i}-1$$

(Equation 5) $$Nomimal\;WACC=\left[1-DF\right]\times \left[\left(1+RROE\right)\left(1+i\right)-1\right]+DF\times \left[\left(1+IR\right)\left(1+i\right)-1\right]\times \left[1-TR\right]$$

(Equation 6) $$Real\;Interest\;Rate\left(IR\right)=\frac{1+d}{1+i}-1$$

ProFinFactor adjusts for project-specific differences in depreciation and tax credits.^64^ This is shown in Equation 7, which is defined as a function of the TR, the present value of depreciation (PVD), and investment tax credit (ITC). Details of the depreciation schedules used to calculate PVD can be found in the NREL 2024 ATB methodology.^16^ In line with NREL ATB 2020 and 2024, an ITC is applied for solar PV and offshore wind, allowing developers to offset 30% of the investment cost from federal taxes.

(Equation 7) $$Project\;Finance\;Factor\left(ProFinFactor\right)=\frac{1-TR\times PVD\times \left(1-\frac{ITC}{2}\right)-ITC}{\left(1-TR\right)}$$

Overnight capital costs (OCC) and grid connection costs (GCC) are measured in $/kW. In addition to CRF and ProFinFactor, the sum of OCC and GCC is multiplied by the construction finance factor (ConFinFactor), the share of total capital costs associated with financing construction of an asset over *C* years, where capital fraction (FC) represents the share of capital used in each year of asset construction and accumulated interest (AI) represents interest expenses on the construction loan (Equation 8). FOM is added to the adjusted capital costs, and the sum is divided by the product of CF and 8760, the number of hours in a year, to convert from $/kW-year to $/kWh (Equation 2). This is multiplied by 1000 to convert to $/MWh, the units of VOM, FUEL, and PTC.

(Equation 8) $$Construction\;Finance\;Factor\;\left(ConFinFactor\right)=\sum_{y=0}^{y=C-1}FC_{y}\times AI_{y}$$

PTC reduce federal tax liabilities during electricity production. In line with NREL ATB 2020, a 24 USD/MWh tax credit is used for onshore wind, while in NREL ATB 2024, a 27.5 USD/MWh tax credit is used, following changes in the IRA. In Equation 9, PTC is defined as the full value of the PTC divided by one minus the tax rate (TR) to adjust for pre-tax LCOE. This is multiplied by the CRF scaled by the 10-year CRF to account for the fact that the PTC is present for only the first 10 years of the project. As a result, the value of the PTC increases in relative terms as WACC increases, with future costs more affected by a higher discount rate.

(Equation 9) $$Production\;Tax\;Credit\;\left(PTC\right)=\frac{PTC_{full}}{\left(1-TR\right)}\times \frac{CRF}{CRF_{10yrs}}$$

We decompose LCOE changes between the NREL ATB 2020 and NREL ATB 2024 models into CAPEX, OPEX, and FINEX to generate Figure 2. To do this, DF and CF are kept constant at levels in the NREL ATB 2024 to isolate changes in LCOE arising from capital and operational costs and changes in WACC. In the NREL 2020 ATB model, as solar PV and onshore wind grid connection costs are not included, we assume grid connection costs are equivalent to those in the NREL 2024 ATB model. In the NREL ATB 2024 model, CF values are not provided for natural gas, so we assume that the values are equal to NREL ATB 2020 figures. For each technology type, NREL provides a range of inputs for different technology specifications. For renewables, we take an average across all specifications. For CCGT, we include only combined cycle gas turbines without carbon capture sequestration. Finally, we adjust values from the NREL ATB 2020 model for 4 years of inflation between 2018 and 2022 to match the base year of the NREL ATB 2024 model. The technology cost assumptions used are provided in Table S4.

To decompose LCOE, we denote the base year as t=0, which is 2018 in the NREL ATB 2020 model, and the future year as t =1, which is 2022 in the NREL ATB 2024 model. WACC inputs from t=0 are denoted as WACC=0, and WACC inputs from t=1 are denoted as WACC=1. When decomposing LCOE, we split Equation 2 into the annualised capital expenditure component (CAPEX) and the annualised operating costs component (OPEX),^65^ shown in Equations 10 and 11.

(Equation 10) $$CAPEX=\frac{\left(CRF\times ProFinFactor\times CAPEX\right)*1000}{CF\times 8760}$$

(Equation 11) $$OPEX=\frac{FOM*1000}{CF\times 8760}+VOM+FUEL$$

To calculate the change in OPEX, we take the difference between t=0 and t=1 and, when relevant, the difference in PTC between t=0 and t=1, assuming WACC remains constant at WACC=0 (Equation 12). To calculate the change in CAPEX, we take the difference between t=0 and t=1, assuming that WACC inputs remain constant at t=0 (Equation 13). To calculate the change in FINEX, we take the difference in CAPEX at t=1 when WACC=0 and WACC=1, and when relevant, the difference in PTC at t=1 when WACC=0 and WACC=1 (Equation 14). PTC is included as values that also depend on WACC. The difference in LCOE between t=0 and t=1 is, therefore, equal to the sum of these differences across CAPEX, OPEX, and FINEX (Equation 15).

(Equation 12) $$\Delta_{OPEX}=\left[OPEX_{t=1}-OPEX_{t=0}\right]+\left[PTC_{t=1,WACC=0}-PTC_{t=0,WACC=0}\right]$$

(Equation 13) $$\Delta_{CAPEX}=CAPEX_{t=1,WACC=0}-CAPEX_{t=0,WACC=0}$$

(Equation 14) $$\Delta_{FINEX}=\left[CAPEX_{t=1,WACC=1}-CAPEX_{t=1,WACC=0}\right]+\left[PTC_{t=1,WACC=1}-PTC_{t=1,WACC=0}\right]$$

(Equation 15) $$LCOE_{1}-LCOE_{0}=\Delta_{OPEX}+\Delta_{CAPEX}+\Delta_{FINEX}$$

To generate Figure 4, we use the inputs from the NREL 2024 ATB in the R&D and Market Scenarios, with the latter including tax credits. To estimate the impact of falls in real interest rates, we keep all other variables constant, including the cost of equity. Here, we break down LCOE into annualised CAPEX and OPEX (Equations 10 and 11). However, we also break out ITC and PTC. For PTC, this is the value from Equation 9. The value of the ITC is calculated as the difference in CAPEX with and without the ITC in the ProFinFactor (Equation 7).

#### Cost competitiveness and changes in WACC by region

To compare the sensitivity of LCOE to WACC by technology and region, we required standardised technology cost assumptions for renewables and fossil fuels. After examining different data sources, we selected the International Energy Agency’s 2024 Global Energy and Climate Model (GEC),^19^ which provides cost assumptions for renewables and fossil fuels in Europe, the U.S., China, and India. Alternatives, such as IRENA, provide country-specific cost assumptions for renewables but not for fossil fuels.

From the IEA GEC model, we extract 2023 base year estimates of capital costs (USD/kW), capacity factor (%), and fuel, CO_2_, and operation and maintenance (OM) costs (USD/MWh). Fuel, CO_2_, and OM reflect the 10-year average from 2023. Grid connection costs are excluded. The IEA GEC model provides estimates for these inputs across three scenarios: the IEA Net Zero Scenario (NZE), which aligns with reaching net zero by 2050 and limiting global warming to 1.5C; the IEA Announced Policies Scenario, which assumes that all government and industry climate commitments are met, and the Stated Policies Scenarios (STEPS); which takes into account current policies, those under development, and planned investment. To be conservative, we use the STEPS scenario, as this is the closest to current operating conditions. For asset lifetimes, we use assumptions from the 2020 IEA Expert Group on Projected Costs of Generating Electricity.^66^

To calculate LCOE, we use a simplified version of Equation 2 shown in Equation 16, as fuel, CO_2_, and OM costs are already measured in USD/MWh. CRF is calculated from real WACC using Equation 3. Tax credits, project finance factors, and construction finance factors are not included, as regional estimates are not provided in the IEA GEC.

(Equation 16) $$Levelised\;Cost\;of\;Electricity\;\left(LCOE\right)=\frac{\left(CRF\times CAPEX\right)*1000}{CF\times 8760}+Fuel,{CO}_{2},OM$$

In Figure 5, we show the impact of a reduction in WACC on the difference in LCOE between renewables and fossil fuels. As the IEA GEC model does not provide exact WACC estimates for each technology by country, we use country-specific WACC estimates in USD from IRENA’s 2023 Power Generation Costs report for solar PV, onshore wind, and offshore wind.^18^ Estimates are provided for the U.S., China, and India. As an estimate is not directly provided for Europe, we calculate a GDP-weighted average of country-level WACC figures for European countries using World Bank GDP data. To generate WACC estimates, IRENA uses a combination of public-private partnership (PPA) transactions, country and technology risk premiums analysis, surveys, and interviews.

IEA and IRENA inputs are shown in Tables S5–S7. As outlined in the main text, we examine four scenarios. In scenario 3, we input a 25% reduction in WACC, after which fossil fuel WACC is 100 bps higher than the riskiest renewable technology. The 100bps figure reflects our results in Table 1 and aligns with the 100bps difference used by NREL. However, while Table 1 focuses on the cost of debt, in scenario 3, we are assuming that the cost of equity for fossil fuels is also 100bps higher and that the 100bps difference in the cost of debt and equity is consistent across regions.

### Quantification and statistical analysis

To empirically compare the spread of renewable transactions (solar and wind) relative to fossil fuel transactions (gas, combined cycle gas turbines, and coal), an ordinary least squares regression is used, as shown in Equation 17. We control for additional transaction-level variables taken from LSEG Data & Analytics. For each transaction *i* occurring in year *t*, we regress spread on loan size in USD million, loan tenor in years, sponsor credit rating, and total project cost in USD million. Sponsor credit rating refers to the Moody’s credit rating of the main project sponsor, with an Aaa rating corresponding to a score of 20, Ca rating corresponding to a score of 1, and a non-rating corresponding to a score of 0. In this context, the sponsor is the group taking the main equity interest in the transaction, often an independent power producer or a utility. In addition, we add country-level control variables from the World Bank, including GDP per capita, inflation, and private credit. Summary statistics and definitions are provided in Tables S1 and S2.

We filter the data only to include transactions where the construction of an asset is being constructed rather than refinancing for an existing asset. The total period analysed is 2000-2024, as country-level economic controls are not all available for 2025 at the time of analysis. In addition, we winsorise spread data at the 2.5% and 97.5% levels to handle outliers. In Equation 17, country fixed effects, currency fixed effects, year fixed effects, and the error term are represented by *θ*_*c*_, *δ*_*u*_, *α*_*t*_, and *ε*_*it*_, respectively. Results are presented as regression coefficients, with robust standard errors shown in parentheses. Statistical significance from t-tests is denoted by ∗p < 0.10, ∗∗ p < 0.05, and ∗∗∗ p < 0.01. See Table 1 for baseline results, and Table S3 for results split by solar and wind.

(Equation 17) $$Spread_{it}=\beta_{0}+\beta_{1}Renewable_{it}+\beta_{2}Loan\;Size_{it}+\beta_{3}Loan\;Tenor_{it}+\beta_{4}Sponor\;Credit\;Rating_{it}+\beta_{5}Total\;Project\;\mathrm{Cos}\;t_{it}+\beta_{6}GDP\;per\;Capita_{ct}+\beta_{7}Inflation_{ct}+\beta_{8}Private\;Credit_{ct}+\theta_{c}+\delta_{u}+\alpha_{t}+\varepsilon_{it}$$

A drawback of project finance data is that spreads are often not reported, especially in developing countries. Given the large proportion of this sample in North America, we also run Equation 1 separately for the U.S. and all other countries. To note, for North America, as shown in Figure S2, the sample is 92% gas within gas & coal. When all regressions are run without coal, the results do not materially change. Given the significant spread changes across time, we also run specification 1 split by 2000–2010 and 2011–2024. We repeat this time split for U.S. and ex-U.S. transactions. Finally, we also split out the renewable assets into solar and wind (Table S3). As coal only accounts for 6% of transactions, we do not split these out from gas.
