# Supplementary material for: Financing costs and the competitiveness of renewable power
Source: iScience. 2025 Oct 15;28(12):113777. doi: 10.1016/j.isci.2025.113777 (PMC12677178; doi:10.1016/j.isci.2025.113777)
Supplement: Document S1. Figures S1–S6 and Tables S1–S8 [file mmc1.pdf]

iScience, Volume *28*

Supplemental information

Financing costs and the competitiveness of renewable power

Christian Wilson, Gireesh Shrimali, and Ben Caldecott

Supplementary Information

Supplementary Figures

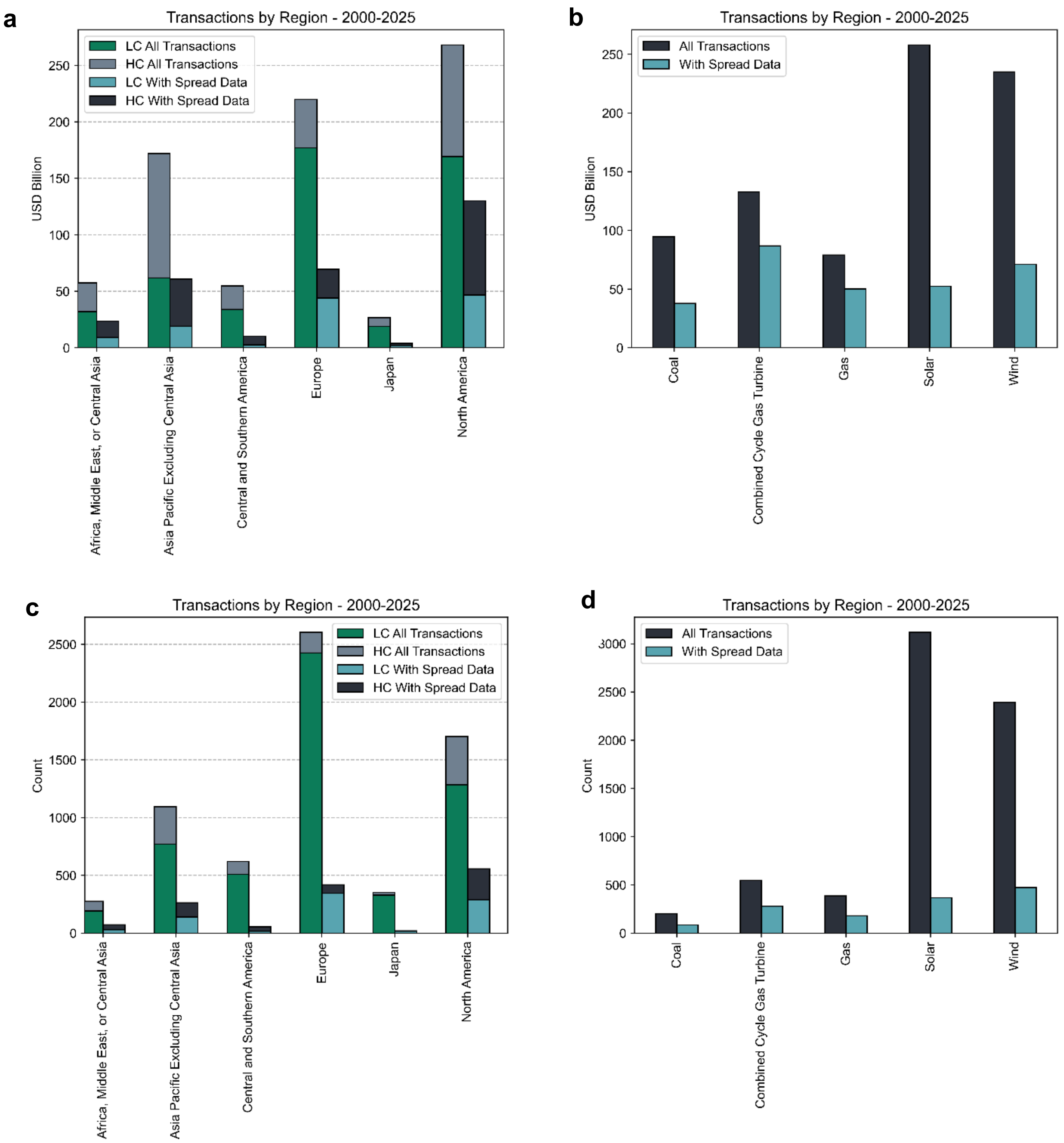

Figure S1. Project finance transactions breakdown by technology and region, related to Figure 1. **a**,**b**, The count of transactions with and without spread data, broken down into region (**a**) and technology (**b**). **c**,**d**, The USD volume of transactions with and without spread data, broken down into region (**c**) and technology (**d**). 2025 data ends in August.

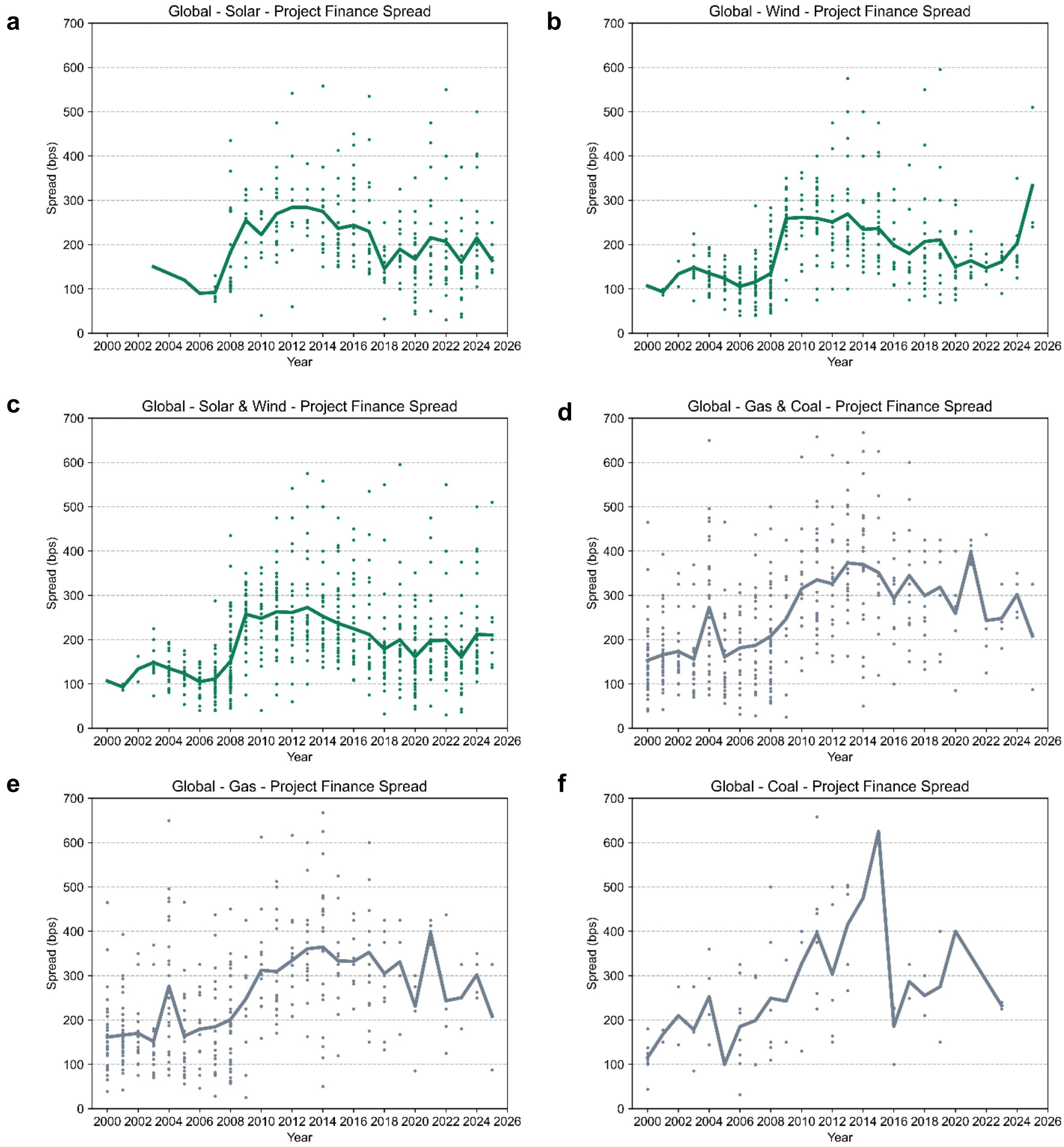

Figure S2. Average spread by technology, related to Figure 1. **a,b,** Average annual spread for solar (**a**) and wind (**b**). **c,d,** Average annual spread for solar & wind **(c)** and gas & coal (**d**). **e,f,** Average annual spread for gas **(e)** and coal (**f**). 2025 data ends in August.

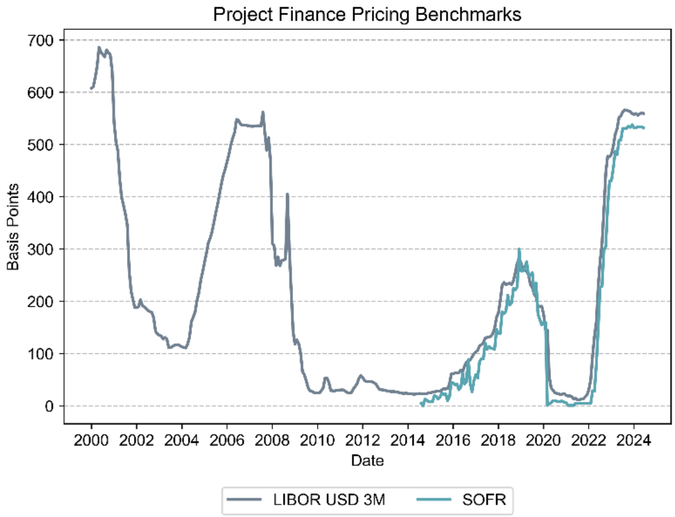

Figure S3. USD LIBOR and SOFR, related to Figure 1. 3-month U.S. Dollar London Interbank Offered Rate (LIBOR) is shown alongside the Secured Overnight Financing Rate (SOFR).

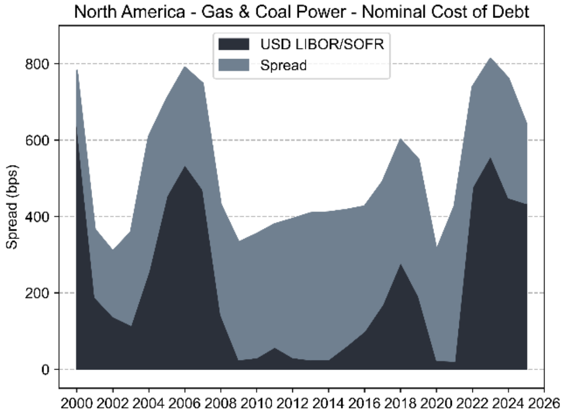

Figure S4. Nominal cost of debt gas & coal spread decomposition, related to Figure 1. Nominal cost of debt for gas & coal project finance transactions in North America, broken down in average annual spread and the floating interest rate (3-month US Dollar London Interbank Offered Rate (LIBOR) and Secured Overnight Financing Rate (SOFR)). 2025 transactions are until August.

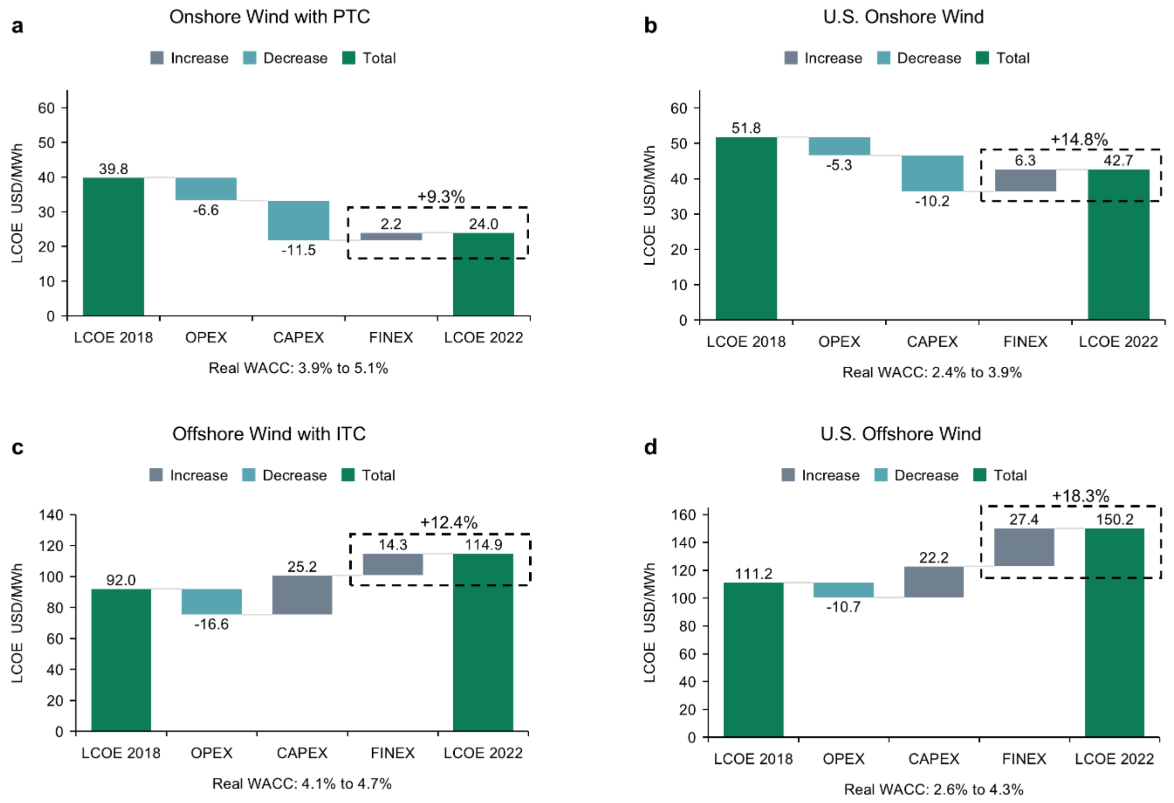

Figure S5. Historical change in LCOE, related to Figure 2. **a**,**b**,**c**,**d**, Change in LCOE between the NREL 2020 ATB model (base year 2018) and the NREL 2024 ATB model (base year 2022) is broken down into the change in OPEX, CAPEX, and FINEX for onshore wind with production tax credits (PTC) (**a**), onshore wind without PTC (**b**), offshore wind with investment tax credits (ITC) (**c**), and offshore wind without ITC (**d**).

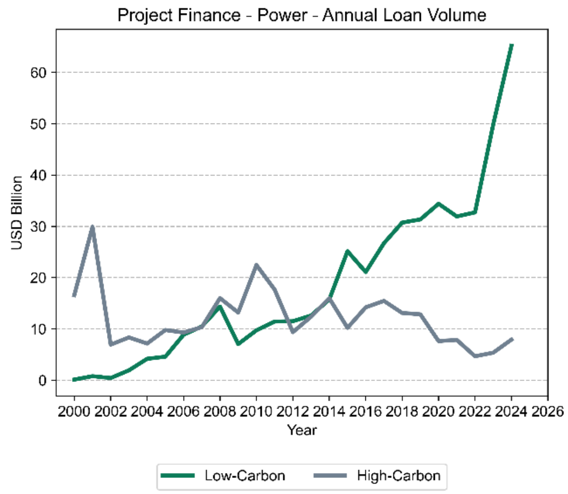

Figure S6. Annual loan volume in USD Billion for low-carbon (solar & wind) and high-carbon (gas & coal) project finance transactions, related to Methods. All transactions are used, not only those with spread data available.

Supplementary Tables

Table S1: Variable definitions, related to Table 1.

Variable	Definition	Source
Spread	The spread on project finance transactions relative to a floating rate benchmark, measured in basis points (bps), equivalent to a hundredth of a 1%.	Eikon
Renewable Asset	Dummy variable equal to one when an asset generates solar or wind power and equal to zero when reliant on gas or fossil fuels. Gas includes assets labelled both as gas and combined cycle turbine.	Eikon
Loan Size	The total sum of loan tranches in USD Million.	Eikon
Sponsor Moody's Credit Rating	The credit rating of the main project sponsor, the group taking the main equity interest in the transaction. We order the credit ratings numerically between 0 and 20.	Eikon
Loan Length	The length of the loan in years.	Eikon
Total Project Cost	The total financing cost of the project in USD Million.	Eikon
GDP per Capita	The gross domestic product (GDP) in USD of the country where the asset is located divided by the population.	World Bank
Inflation	Percentage inflation, as measured by the GDP deflator.	World Bank
Access to Credit	Domestic credit provided to the private sector as a percentage of GDP.	World Bank

Table S2: Project finance transactions summary statistics, split by technology type, related to Table 1.

Renewable Transactions	N	Mean	Std. Dev.	Min	Max
Spread	812	202.520	105.078	43.750	616.667
Renewable Asset	812	1.000	0.000	1.000	1.000
Loan Size	812	147.019	195.726	1.557	2802.542
Sponsor Moody's Rating	788	0.347	1.908	0.000	20.000
Loan Length	812	10.406	5.845	0.000	33.000
Total Project Cost	812	400.502	737.932	8.053	11210.167
GDP per Capita	773	40496.882	17476.798	618.398	66682.617
Inflation	773	2.611	3.742	-12.031	49.196
Access to Credit	743	143.930	48.785	14.478	220.316

Fossil Fuel Transactions	N	Mean	Std. Dev.	Min	Max
Spread	499	521	260.502	141.880	43.750
Renewable Asset	499	521	0.000	0.000	0.000
Loan Size	499	521	322.455	360.140	14.500
Sponsor Moody's Rating	486	508	1.258	3.598	0.000
Loan Length	499	521	10.194	5.376	0.000
Total Project Cost	499	521	866.029	1026.220	14.500
GDP per Capita	496	515	37864.640	21212.620	641.289
Inflation	496	515	3.863	5.736	-16.559
Access to Credit	478	490	129.570	62.783	8.285

Table S3: OLS regression results limited to solar or wind, related to Table 1. When comparing results for only wind assets to gas & coal assets, we observe significant relationship for the wind asset dummy across all specifications, except for ex-U.S. in the 2000-2020 time period. For solar assets only, we observe a weaker relationship. Over the full solar sample, we see a negative relationship significant at the 1% level. However, when broken down by region, this only holds in the 2011-2024 time period. This is due to a positive, rather than negative relationship observed for ex-U.S. in the 2000-2010 time period, which offsets the negative relationship in the U.S. significant at the 1% level.

		2000-2010			2011-2024		
	All Transactions	All	US	ex-US	All	US	ex-US
Panel A	1	2	3	4	5	6	7
Wind	-88.328*** (8.503)	-61.477*** (12.492)	-108.438*** (13.193)	-7.531 (20.989)	-122.204*** (13.527)	-115.675*** (11.025)	-92.213*** (31.015)
Year FE	Yes	Yes	Yes	Yes	Yes	Yes	Yes
Country FE	Yes	Yes	Yes	Yes	Yes	Yes	Yes
Currency FE	Yes	Yes	Yes	Yes	Yes	Yes	Yes
Asset-Level Controls	Yes	Yes	Yes	Yes	Yes	Yes	Yes
Country-Level Controls	Yes	Yes	Yes	Yes	Yes	Yes	Yes
N	885	474	176	298	411	315	223

		2000-2010			2011-2024		
	All Transactions	All	US	ex-US	All	US	ex-US
Panel B	1	2	3	4	5	6	7
Solar	-81.565*** (12.386)	60.726 (40.315)	-80.465*** (23.864)	74.013 (45.521)	-104.496*** (12.265)	-121.420*** (13.131)	-76.036** (31.790)
Year FE	Yes	Yes	Yes	Yes	Yes	Yes	Yes
Country FE	Yes	Yes	Yes	Yes	Yes	Yes	Yes
Currency FE	Yes	Yes	Yes	Yes	Yes	Yes	Yes
Asset-Level Controls	Yes	Yes	Yes	Yes	Yes	Yes	Yes
Country-Level Controls	Yes	Yes	Yes	Yes	Yes	Yes	Yes
N	791	337	112	225	454	253	201

*$p < 0.10$, ** $p < 0.05$, *** $p < 0.01$ denote statistical significance at the 10%, 5% and 1% level, respectively. Robust standard errors are shown in parentheses.

Table S4: NREL Technology Cost Assumptions, related to Figure 2 and 4. To note, as discussed in the methods, certain 2018 inputs are adjusted for inflation (operational, overnight, grid connection, variable, and fuel costs), or held constant at 2022 levels (debt fraction and capacity factor).

	2018	2022
Solar PV with ITC		
Capital recovery period (years)	30	30
Inflation	2.50%	2.74%
Cost of debt (real)	1.46%	4.15%
Cost of equity (real)	5.12%	5.61%
Debt fraction	52.10%	52.10%
WACC real	2.69%	3.93%
Operational cost	22.13	24.00
Overnight costs	1824.28	1366.60
Grid connection costs	75.72	65.00
Capacity factor	26.00%	26.00%
Tax rate	25.70%	25.70%
CRF	4.90%	5.74%
CFF	1.02	1.04
ITC	30.00%	30.00%
PFF	0.69	0.70
Solar PV		

Capital recovery period (years)	30	30
Inflation	2.50%	2.50%
Cost of debt (real)	1.46%	4.39%
Cost of equity (real)	5.12%	6.83%
Operational cost	22.13	24.00
Overnight costs	1824.28	1366.60
Grid connection costs	116.49	100.00
Capacity factor	28.00%	28.00%
Tax rate	25.70%	25.70%
CRF	4.28%	5.43%
PFF	1.04	1.05
CFF	1.02	1.04
Onshore wind with PTC		
Capital recovery period (years)	30	30
Inflation	2.50%	2.74%
Cost of debt (real)	1.46%	4.15%
Cost of equity (real)	6.34%	6.09%
Operational cost	50.09	33.00
Overnight costs	2183.07	1554.00
Grid connection costs	116.49	100.00
Capacity factor	41.00%	41.00%
Tax rate	25.70%	25.70%
CRF	5.82%	6.25%
PFF	1.06	1.06
CFF	1.04	1.06
PTC	24.00	27.50
Onshore Wind		
Capital recovery period (years)	30	30
Inflation	2.50%	2.50%
Cost of debt (real)	1.46%	4.39%
Cost of equity (real)	6.34%	6.34%
Operational cost	149.11	91.00
Overnight costs	3529.73	4774.00
Grid connection costs	1249.97	1446.00
Capacity factor	39.90%	40.00%
Tax rate	25.70%	25.70%
CRF	4.85%	5.97%
PFF	1.04	1.06
CFF	1.07	1.11
Offshore wind with ITC		
Capital recovery period (years)	30	30
Inflation	2.50%	2.74%
Cost of debt (real)	1.46%	4.15%
Cost of equity (real)	7.32%	7.55%
Operational cost	149.11	91.00
Overnight costs	3529.73	4774.00
Grid connection costs	1249.97	1446.00
Capacity factor	40.00%	40.00%

Tax rate	25.70%	25.70%
CRF	5.64%	6.44%
CFF	1.07	1.11
ITC	30.00%	30.00%
PFF	0.69	0.70
Offshore wind		
Capital recovery period (years)	30	30
Inflation	2.50%	2.50%
Cost of debt (real)	1.46%	4.39%
Cost of equity (real)	7.32%	7.80%
Operational cost	149.11	91.00
Overnight costs	3529.73	4774.00
Grid connection costs	1249.97	1446.00
Capacity factor	39.90%	40.00%
Tax rate	25.70%	25.70%
CRF	4.85%	5.97%
PFF	1.045	1.057
CFF	1.075	1.109
CCGT		
Capital recovery period (years)	20	20
Inflation	2.50%	2.74%
Cost of debt (real)	2.44%	5.12%
Cost of equity (real)	7.32%	7.55%
Operational cost	15.14	36.00
Overnight costs	1216.18	1335.00
Grid connection costs	116.49	100.00
Capacity factor	55.00%	55.00%
Tax rate	25.75%	25.75%
Variable cost	2.33	2.29
Fuel cost	20.97	18.60
CRF	7.32%	8.10%
PFF	1.13	1.15
CFF	1.05	1.12

Table S5: IEA 2024 Global Energy and Climate Model technology cost assumptions, related to Figure 5.

	Capital costs (USD/kW)	Capacity factor (%)	Fuel, CO_2, O&M (USD/MWh)
United States			
Nuclear	5 000	90	30
CCGT	1 000	55	35
Solar PV	1 110	20	10
Wind onshore	1 500	42	10
Wind offshore	4 060	41	35
European Union			
Nuclear	6 600	70	35
CCGT	1 000	20	130
Solar PV	750	14	10
Wind onshore	1 630	29	15
Wind offshore	3 120	50	15
China			
Nuclear	2 800	80	30
Coal	800	55	55
Solar PV	670	13	10
Wind onshore	990	24	10
Wind offshore	2 380	32	20
India			
Nuclear	2 800	75	30
Coal	1 200	70	40
Solar PV	710	20	5
Wind onshore	1 210	26	15
Wind offshore	2 620	33	25

Table S6: IEA Expert Group on Projected Costs of Generating Electricity asset lifetime assumptions, related to Figure 5.

Technology	Lifetime in years
Solar PV	25
Onshore wind	25
Offshore wind	25
Gas-fired power plant	30
Coal-fired power plant	40
Nuclear power plant	60

Supplementary S7: IRENA Cost of Capital Benchmarks, related to Figure 5. Europe is not provided directly by IRENA, so a GDP-weighted average of individual country WACC estimates is calculated.

IRENA 2023 Real WACC Estimate	Solar PV	Onshore Wind	Offshore Wind
US	5.87%	4.40%	5.87%
Europe	4.20%	4.38%	5.45%
China	3.04%	3.04%	6.12%
India	8.41%	8.41%	NA

Supplementary S8. Data and code availability.

Data	Source
Project finance transactions	At the time of analysis, Refinitiv Eikon was used. As of 30th June 2025, Eikon has been discontinued and transferered to LSEG workspace https://www.lseg.com/en/data-analytics/products/workspace. Data is available under licence agreement with LSEG Data & Analytics.
Country-level control variables	Publically available data from the World Bank https://databank.worldbank.org/.
U.S. LCOE cost components	NREL data for the ATB 2024 model is publicly available at https://atb.nrel.gov/electricity/2024/data and for the ATB 2020 model at https://atb-archive.nrel.gov/electricity/2020/data.php
Europe, U.S., China, and India LCOE cost components	IEA GEC model input data is publicly available at https://www.iea.org/data-and-statistics/data-product/global-energy-and-climate-model-key-input-data
Country and technology specific WACC	IRENA WACC data is publicly available at https://www.irena.org/Publications/2024/Sep/Renewable-Power-Generation-Costs-in-2023
